# Development of energy‐rich protein bars and in vitro determination of angiotensin I‐converting enzyme inhibitory antihypertensive activities

**DOI:** 10.1002/fsn3.2756

**Published:** 2022-01-23

**Authors:** Sidra Jabeen, Faiqa Javed, Navam S. Hettiarachchy, Amna Sahar, Aysha Sameen, Moazzam Rafiq Khan, Azhari Siddeeg, Ayesha Riaz, Rana Muhammad Aadil

**Affiliations:** ^1^ National Institute of Food Science and Technology University of Agriculture Faisalabad Pakistan; ^2^ Department of Food Science University of Arkansas Fayetteville Arkansas USA; ^3^ Department of Food Engineering University of Agriculture Faisalabad Pakistan; ^4^ Department of Food Engineering and Technology Faculty of Engineering and Technology University Gezira Wad Medani Sudan; ^5^ Institute of Home Sciences University of Agriculture Faisalabad Pakistan

**Keywords:** ACE‐I activity, cheese, energy‐rich protein bars, peptides, proteins

## Abstract

Three energy‐rich protein (ERP) bars were prepared to meet the daily recommended dietary allowance (RDA) for the protein of Pakistani athletes. The bars were developed using dates, cheddar cheese (CC), whey protein isolate (WPI), roasted chickpea flour, and rice flour in different proportions. Bar #1 contained 64 g dates, 16 g dried apricots, 12 g WPI, and 8 g ripened CC. Bar #2 contained the same proportion of these ingredients with an addition of 12.5 g roasted chickpea flour, while bar #3 contained 6.25 g roasted rice and 6.25 g roasted chickpea flour. All the ingredients were homogeneously mixed into paste to form bars weighing 100–110 g per serving size. These bars were studied for the compositional analysis (moisture, protein, and lipid content), protein characterization through sodium dodecyl sulfate–polyacrylamide gel electrophoresis (SDS‐PAGE), and in vitro determination of the angiotensin I‐converting enzyme (ACE‐I) antihypertensive activity. Moisture and lipid content in bars were 22% and 0.057%–0.313%, respectively, while protein, fiber, and ash contents varied from 22.3% to 23.6%, 6.66 to 5.81, and 2.12% to 2.44%, respectively. The minimum energy content was recorded (272.70 Kcal/100 g) in bar #1 while bar #3 showed the highest energy content 274.65 Kcal/110 g with the addition of (5%) roasted chickpea and rice flour, respectively. Electrophoresis analysis of proteins in bar # 1 (cheese +WPI) showed the four bands at 62, 24, 20, and 12 kDa. Bar #2 (10% roasted chickpea flour) showed some additional bands at 40, 36, 34, and 28 kDa while relatively lower antihypertensive activity than bars #1 and 3. The study revealed that adding 10% roasted chickpea flour (bar #2) increased the protein content and diversity in proteins. It provided 40% proteins to athletes and could be helpful to meet their R.D.A. by consuming two bars/day.

## Graphical abstract

These bars were studied for the compositional analysis (moisture, protein, and lipid content), protein characterization through sodium dodecyl sulfate–polyacrylamide gel electrophoresis (SDS‐PAGE), and in vitro determination of the angiotensin I‐converting enzyme (ACE‐I) antihypertensive activity. Moisture and lipid content in bars were 22% and 0.057%–0.313%, respectively, while protein contents varied from 22.3% to 23.6%. Electrophoresis analysis of proteins in bar # 1 (cheese +WPI) showed the four bands at 62, 24, 20, and 12 kDa. Bar #2 (10% roasted chickpea flour) showed some additional bands at 40, 36, 34, and 28 kDa while relatively lower antihypertensive activity than bars #1 and 3.

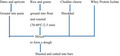

## 1 INTRODUCTION

The growing concern for health and fitness has raised consumer demand for biologically active food products and driving the market for nutrient‐dense bars for athletes, soldiers, teenagers, and school‐aged children. These bars are an immediate energy source, meal replacement bars for increased muscle mass and weight management. An immense range of food products helps improve cognition ability, enhance physical output and muscular performance, increase endurance, and much more (Jabeen et al., 2021; Knapik et al., 2016). Among these products, the growth rate of protein‐rich products is very high, including whey protein isolate (WPI) and soy protein (Keogh et al., 2019).

Dairy products such as cheddar cheese (CC) and WPI were rich sources of proteins and bioactive peptides. The angiotensin converting enzyme can regulate blood pressure (BP) by converting angiotensin I into vasoconstrictor angiotensin II. Milk‐derived peptides have the potential to inhibit the angiotensin converting enzyme and lower BP. The average activity is ACE‐I peptides about 0.79 times greater than the average activity of captopril (Martin‐del‐Campo et al., 2019).

Keeping in mind the safety concerns of food supplements, the consumption of ERP bars is a healthy choice for athletes and other consumers. In the formulation of ERP bars, dates, dried apricots, CC, WPI, chickpea, and rice flour make ingredients' physical and chemical interaction. Fruits are being used to provide a plentiful supply of nutrients and functional components. In contrast, the addition of fiber‐rich components (such as chickpea flour) increased its water absorption by delaying the process of hydration (Rawat & Darappa, 2015). Thus, adding flour (chickpea and rice) provides textural properties and shelf stability to ERP bars (Jimenez‐Moreno et al., 2020).

Pakistan ranked at 7th and 10th positions in the production of dates and apricots, respectively (Kousar et al., 2019). The export of these fruits is often limited; about 40%–45% of their total production will be wasted due to poor supply chain, handling, and value addition (Abul‐Soad et al., 2015). Dates and apricot are available at lower prices in Pakistan and utilized as a raw material to formulate different food products. These fruits are highly appreciated in the fruit markets of Pakistan owing to their taste and aroma. The dates contain a considerable amount of carbohydrates 73.5%, ash 1.5%, proteins 2.3%, lipids 0.2%, and vitamins and minerals (Rashwan et al., 2020; Younas et al., 2020). Polysaccharides in dates work as functional components and provide bioactive compounds. The dried apricots are rich in carbohydrates, β‐carotenes, water‐soluble vitamins (vitamin C, thiamine, pantothenic acid, and niacin), and vitamins A and K. It also contains a substantial amount of organic acids, volatile compounds, terpenoids, and esters (Ghnimi et al., 2018; Shehzad et al., 2021; Younas et al., 2020).

Sustaining a healthy diet for athletes to supplement their caloric intake and basic nutrients is important for providing intense exercise and optimizing their immune body functions (Indoria & Singh, 2016). The recommended dietary allowance (RDA) guidelines for athletes recommended the quantity of carbohydrates and protein up to 7–12 g and 1.2–2.0 g/kg body weight, respectively, and fat intake 20%–35% of total calories per day (Jager, 2017). In Pakistan, the least importance is given to the diet of athletes, the only food which is preferred for them by their instructors and nutritionists is whey protein isolate. But now, with increasing the awareness of nutrition, the people of Pakistan are more concerned about their diet. Keeping in view the health significance of dates, apricot, cheese, WPI, chickpea, and rice flours, the project was designed to develop nutri‐bars for the athletes. Therefore, the objective was to introduce a new product that meets the R.D.A. for Pakistani athletes by consuming a serving size of two bars/day to investigate the composition, protein fragments (SDS‐PAGE), and angiotensin I converting enzyme inhibitory (ACE‐I) antihypertensive activity in ERP bars.

## 2 MATERIALS AND METHODS

### 2.1 Materials

The ingredients of ERP bars (dates, dried apricots, 6‐month ripened CC, chickpea gram, and rice flours) were purchased from local grocery stores in Fayetteville, Ark., U.S.A. WPI (95% pure) was obtained from Glanbia Nutritionals, Inc. (Thermax ^R^ 690). The chemicals for determining protein content, protein extraction, and ACE‐I inhibitory activity were purchased from V.W.R. International, Inc. and Sigma‐Aldrich Co. The chemicals for electrophoresis were procured from Bio‐Rad Laboratories, Inc. All the other reagents used were of analytical grade and acquired from V.W.R. International, Inc.

### 2.2 Preparation of ERP bars

**FIGURE 1:**
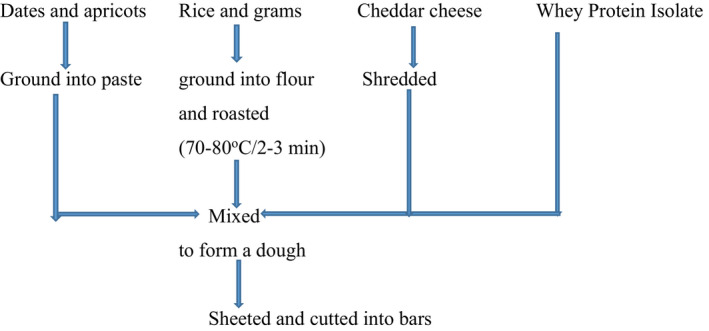
Flow diagram of ERP bars

Three different formulations of ERP bars were prepared and designated as #1, #2, and #3 (Table 1). After cleaning and soaking, the dates and dried apricots were ground into a paste. The CC was shredded, while chickpea and rice flours were roasted (80°C/3 min) (Figure 1). All these ingredients and WPI were mixed homogeneously to form the dough, followed by sheeting, and cutting into bars with length 6 cm, width 2 cm, and depth 1 cm. Bar #1 contained 64 g dates, 16 g dried apricots, 12 g WPI, and 8 g ripened CC. Bar #2 contained the same proportion of these ingredients with an addition of 12.5 g roasted chickpea flour, while bar #3 contained 6.25 g roasted rice and 6.25 g roasted chickpea flour.

**TABLE 1:** Formulations of ERP bars

Product #	Dates (g)	Apricots (g)	WPI (g)	Cheese (g)	Gram flour (g)	Rice flour (g)	Total (g)	Calories
1	64	16	12	8	—	—	100	272.70
2	64	16	12	8	10	—	110	274.26
3	64	16	12	8	5	5	110	274.65

Note

The formulations were designed keeping in view the requirement of athletes.

### 2.3 Compositional analysis

The composition analysis (moisture, protein, lipid, fiber, and ash contents) of ERP bars was determined according to the 930.15, 930.29, 991.36, 991.43, and 942.05, respectively, standard methods of the Association of Official Analytical Chemists (AOAC, 2012). The results were expressed on a dry‐weight basis. Water activity (A_w_) was also determined using an A_w_ meter (AquaLab).

### 2.4 Calorific value

The calorific value of ERP bars (Kcal per gram) was determined by using Oxygen Bomb Calorimeter (IKA‐WERKE, GMBH and CO) according to the procedure of Younas et al. (2015).

### 2.5 Preparation and characterization of protein/peptide extract from ERP bars

To prepare and characterize protein extracted from the bars, ERP bars were freeze dried and ground to powder (80‐mesh, 0.2 mm) using a laboratory grinder (IKA WERKE grinder model M20, IKA. Works, Inc.). It was then mixed with hexane (sample powder:hexane ratio was 1:3, w/v) with continuous stirring at ambient temperature for 3–4 h. The hexane was separated by vacuum filtration, and defatted samples were dried under a hood at ambient temperature for 24 h to remove the residual hexane. Deionized water (DI) was added to the defatted sample (1:10, w/v), and the dispersion was stirred for 2 h and centrifuged (Model J2‐21, California, U.S.A.) at 10,000 × *g* for 30 min at 4°C. The insoluble protein was precipitated to its isoelectric point at pH 4.6 using 1 M HCl solution with the centrifugation at 10,000 × *g* for 30 min at 4°C. The isoelectrically precipitated protein was reconstituted with DI water and adjusted its pH 7.0 with 1 M NaOH solution. The insoluble and isoelectrically precipitated reconstituted protein was freeze dried and stored at 4°C, subjected to electrophoresis, and then evaluated for their in vitro antihypertensive (ACE‐I) activities.

### 2.6 Characterization of proteins in ERP bars by electrophoresis

The molecular size was determined by following the method of Laemmli (1970). The sodium dodecyl sulfate–polyacrylamide gel electrophoresis (SDS‐PAGE) was used with 4% stacking slab gel and 17% resolving gel. The protein solutions were applied on SDS‐tris‐glycine discontinuous buffer system with a constant electric field of 40 mA. Ten microliters of protein solution of each bar sample (10 mg/ml ERP bar protein) extract dissolved in reducing buffer (10% glycerol, 10% SDS, 62.5 mM Tris HCl with 6.8 pH, 0.01% bromophenol blue, and 0.5% 2‐mercaptoethanol) system was loaded onto the gel. The electrophoresis was performed for 90–120 min with a constant power supply (Bio‐Rad Laboratories). The molecular size of proteins in standard marker solution was in the broad range of 6.5–200 kDa (200 kDa myosin, 116.25 kDa β‐galactosidase, 97.4 kDa phosphorylase b, 66.2 kDa serum albumin, 45 kDa ovalbumin, 31 kDa carbonic anhydrase, 21.5 kDa trypsin inhibitor, 14.4 kDa lysozyme, and 6.5 kDa aprotinin). Coomassie brilliant blue (0.1% in acetic acid/ethanol/water solution 10/40/50, v/v/v) was used as the staining solution of the slab gel to identify each protein's molecular size in bar samples.

### 2.7 Determination of ACE‐I inhibitory activity

Angiotensin I‐converting enzyme inhibitory activities were determined by following the method of Horax et al. (2017). Fifty microliters protein extract solution (10 mg/ml in 0.1 M phosphate buffer pH 8.3), 50 µl ACE‐I solution (125 mU/ml in 0.1 M phosphate buffer pH 8.3), and 150 µl HHL (hip‐puryl‐L‐histidyl‐L‐leucine) solution (12.5 mM in 0.1 M phosphate buffer pH 8.3 containing NaCl of 0.5 M) were incubated at 37°C for 1 h. The reaction was stopped by adding 250 µl of 1 N HCl. For negative control, 1 N HCl was added into 50 µl phosphate buffers before adding the enzyme. One milliliter ethyl acetate was used to extract hippuric acid liberated from H.H.L. by ACE‐I activity and centrifuged for 10 min. An aliquot of ethyl acetate (750 µl) was heated at 90°C up to dryness, and the residue was dissolved in 1 ml DI water. The absorbance (Abs) of the liberated hippuric acid was recorded spectrophotometrically at 228 nm, and the inhibitory activity (%) was calculated according to the following formula:

$$\%\;\text{Inhibitory activity}=\frac{1‐\left(\text{Abs of sample}‐\text{Abs of negative control}\right)}{\text{Abs without sample}‐\text{Abs of negative control}}\times 100$$

### 2.8 Sensory analysis

Sensory analysis was conducted in the Sensory Service Centre, University of Arkansas (Fayetteville, AK, U.S.A). Sensory evaluation was conducted at ambient temperature (20–25°C) in well‐ventilated seating compartments (cabins), with a good lighting facility. Twenty‐five panelists, including teachers (5 males and 5 females) and students (13 males and 12 females, age 20–25 years) within the department, took part in sensory evaluation. Each panelist was offered drinking water during evaluation to rinse the mouth before testing the next bar. The product attributes to be tested, including taste, aroma, texture, color, and overall acceptability, were based on a 9‐point hedonic scale ranging from "1 dislike‐extremely to 9 like‐extremely" (Jabeen et al., 2020). Based on sensory testing, the selected ERP bars were analyzed for composition, protein fractions, and ACE‐I potential.

### 2.9 Statistical analysis

The results are expressed as means ±standard deviation of triplicate determinations. The data were analyzed through analysis of variance (ANOVA) to compare means to evaluate the level of significance at 5%. Tukey's test was used for multiple comparisons between means (α = 0.05). Simple linear correlation analysis was used to determine a relationship between the mean values of total peptides and their bioactivities.

## 3 RESULTS AND DISCUSSION

### 3.1 Moisture and water activity (A_w_)

Moisture content and A_w_ are important factors that determine the shelf stability of food. It has a strong relationship with the growth of microorganisms and the texture of food products (Rahman and Labuza, 2020). The compositional analysis of ERP bars is presented in Table 2. There was a nonsignificant difference (*p* > .05) in moisture content of all ERP bars (22.5% ± 0.1%). The highest A_w_ was noted in bar #1, whereas the lowest A_w_ was found in bar #2. Results pertaining to change in A_w_ are in line with the findings of Estevez et al. (2000), who studied that A_w_ level decreased from 0.71 to 0.52 at 0 and 60 days, respectively, in cereal and nut bars during storage. The reason was the addition of two levels of peanut or walnut (15% and 18%) in the formulated cereal bars.

**TABLE 2:** Compositional analysis of ERP bars

Product #	Water activity (A_w_)	Moisture (%)	Protein (%)	Lipid (%)	Crude fiber (%)	Crude ash (%)	NFE (%)
1	0.53 ± 0.01^a^	22 ± 0.01^a^	22.31 ± 0.2^a^	0.313 ± 0.02^a^	6.66 ± 0.11^b^	2.12 ± 0.07^b^	28.35 ± 0.05^c^
2	0.51 ± 0.01^a^	22 ± 0.01^a^	23.6 ± 1.0^a^	0.057 ± 0.04^c^	7.16 ± 0.04^a^	2.44 ± 0.03^a^	34.33 ± 0.03^b^
3	0.51 ± 0.02^a^	22 ± 0.01^a^	23.2 ± 0.0^a^	0.21 ± 0.03^b^	5.81 ± 0.02^c^	2.32 ± 0.03^a^	35.36 ± 0.04^a^

Note

Values are means ±standard deviation of three replications. Means with different letters within each column represent a significant difference at *p* < .05.

For the development of the date bar, whey protein was used as dates do not provide a good amount of protein. The ingredients used for the formulation of the dates bar were very low in moisture content compared to other ingredients. However, dry roasting of ingredients helps improve the shelf stability of the product without affecting the sensory profile (Rajni et al., 2012).

A nonsignificant effect of moisture and A_w_ could be due to the addition of ingredients like dates, dried apricots, and CC, which contributed to the prepared bars' moisture content. In a study conducted on the development of nutritious cereal bars and evaluated for A_w_ results showed that all bars have low A_w_ levels (~0.470). Apple, pear, and date coproducts could be used successfully as food ingredients for different formulations of cereal bars (Bchir et al., 2018). A study conducted on the development of apricot date bars indicated that A_w_ ranged from 0.534 to 0.546, and A_w_ of bars was affected significantly with the addition of dried paste of apricots. Moisture was also significantly affected, ranging from 17.14% to 19.21% (Nadeem et al., 2012a, 2012b).

In the present study, a relatively higher A_w_ in bar #1 is due to the higher moisture content of dates and apricot. At the same time, the addition of chickpea and rice flours in bars #2 and #3 could have contributed toward the reduction in A_w_ due to the use of dry roasted ingredients. Barbosa‐Canovas et al. (2020) reported that food products with A_w_ lower than 0.7 have a good shelf life and are stable for approximately 6 months. The low A_w_ of the bars (# 2 and 3) could be due to moisture loss during the roasting of gram flour. Low A_w_ showed a low risk of pathogenic spoilage, microbial growth, and good product shelf stability. Moreover, the addition of chickpea flour increased the water absorption in the product (Sofi et al., 2020). It is possible that the addition of roasted rice and gram flour contributed to the reduction in moisture and A_w_ of ERP bars.

### 3.2 Lipid content

Fat is considered an important part of daily diet manipulation. Its moderate amount in food has a good impact on athletic exercise, metabolism, and supportive effect on body composition and recovery (Witard et al., 2019). The lipid content in bars ranged from 0.21 ± 0.03% to 0.057 ± 0.04%. The lipid content was significantly (*p* < .01) lower in bars #2 (contained 10% g flour) and #3 (contained 5% g flour and 5% rice flour) as compared to bar #1 (contained ripened cheese as the only source of fat). The results showed that the addition of chickpea gram flour up to 10% in ERP bars helped to decrease the lipid content (0.313 ± 0.02%–0.057 ± 0.04%), while the addition of 5% rice flour decreased the lipid content from 0.313% ± 0.02% to 0.21% ± 0.03%, respectively. Likewise, USDA Food Composition Database revealed that chickpea's moisture and protein content were 10.8% and 22.3% (USDA, 2018). It is reported that chickpea flour contains about 1.80% fat, 20.21% protein, and 1.8% ash (Dandachy et al., 2019). Cheese is present in all three bars, which contained 53.4% ± 0.02% fat and the source of saturated fatty acids that are less prone to oxidative reactions. Dairy fat also contains fat‐soluble vitamins A, D, E, and K, in which vitamins A and E are considered important fat‐soluble antioxidants to protect the food from rancidity (Khan et al., 2019).

### 3.3 Protein content

In ERP bars, WPI and CC were used as the main source of protein. However, the addition of roasted chickpea and rice flour enhanced their sensory and textural properties. The average protein content in the three ERP bars # 1, 2, and 3 were 22.3% ± 0.2%, 23.6% ± 1%, and 23.2% ± 0%, respectively, in 100–112 g serving size that was non‐significant (*p* > .05) among all three ERP bars. In bar #1, CC and WPI were the major protein sources. The addition of 10% roasted chickpea flour increased the protein content in bar #2 (23.6% ± 1%) because roasted chickpea flour contained 20.21% protein. In comparison, the addition of 5% chickpea and 5% rice flour increased the protein content in bar #3 (23.2% ± 0%), which is slightly lower than bar #2. This could be due to lower protein in rice flour (6.25%) than chickpea flour. The addition of roasted chickpea gram flour in ERP bars contributes substantially to the increased protein content since it is an excellent source of protein, carbohydrates, several water‐soluble vitamins, and minerals (El‐Beltagi et al., 2017).

### 3.4 Fiber content

The data regarding crude fiber differed significantly among different nutri‐bars (Table 2). The maximum mean value was observed in bar #2 (7.16 ± 0.04) and minimum in bar #3 (5.81% ± 0.02%). It is evident from the results that there was a decrease in fiber content of newly formulated ERP bar due to the addition of apricot paste. In a study conducted on the development and physicochemical characterization of apricot–date bars, the crude fiber was noted in the range 5.55–6.14 g/100 g in the developed four products (Nadeem et al., 2012a, 2012b). Zahra et al. (2020) explicitly reported the composition of date bars by using local ingredients such as date paste, roasted chickpea, roasted white oat, skim milk, and dark chocolate formulating 15 products and reporting the content of crude fiber from 2.20% to 4.24%. In another study by Zahra et al. (2014), the nutri‐bars were prepared by using the combinations of dried apricots, dried milk powder, and chickpea flour and reported a fiber content of 5.2%. Munir et al. (2016) developed the date bars and reported the fiber content as 3.48%–4.36% that strengthened the findings of the present study.

### 3.5 Ash content

The crude ash was lowest in bar #1 (2.12% ± 0.07%) and the highest amount was noted in bar #2 (2.44% ± 0.03%). In a study conducted on development and physicochemical characterization of apricot–date bars, maximum value for ash (4.20 g/100 g) was observed in the bar containing 30 g apricot (T4) against minimum in T1 (4.06 g/100 g) on a dry weight basis. This study demonstrated that the use of dried apricots and dates to formulate the ERP bars can provide a significant amount of dietary fiber and ash (Nadeem et al., 2012a, 2012b). Zahra et al. (2014) reported in their study that the ash content of nutri‐bars was increased (3.50% ± 0.02%) as the apricot content was increased (12 g). In a study conducted on bars made with cereal combinations of dried fruit in different combinations, ash content was recorded from 1.15% to 1.38% (Guimaraes & Silva, 2009).

### 3.6 Nitrogen free extract (NFE)

Mean values for nitrogen‐free extract of nutri‐bar (Table 2) showed the lowest NFE in bar #1 (28.35% ± 0.05%) developed with 64 g dates, 16 g dried apricot, 12 g WPI, and 8 g CC, whereas the highest value (35.36% ± 0.04%) was recorded in bar #3 containing the additional amount of roasted chickpea flour (5 g) and rice flour (5 g). Results showed that the NFE content increased with the addition of chickpea and rice flour. Zahra et al. (2014) reported the highest values of NFE contents in their products that contained higher amounts of cereals and dried apricot. Nadeem et al. (2012a); Nadeem et al. (2012b) prepared the apricot date bar with a combination of gram flour and skim milk powder. They reported the higher NFE content in the product that contained a higher concentration of dried apricot (30 g) with the addition of 12 g gram flour. Agbaje et al. (2016) prepared the granola with the addition of puffed glutinous rice. They reported that the NFE (carbohydrate) content of the product was affected by increasing the fiber content. The product with the lower value of crude fiber had higher carbohydrate content, which reflects the energy contents of the cereal bar/granola. Similar results were found in the current study, the bar with higher fiber content (6.66% ± 0.11%) showed lower value of carbohydrate (28.35% ± 0.05%).

The results regarding the energy content of ERP bars showed the highest energy content in bar #3, that is, 274.65 Kcal/110 g, while bars #1 and 2 contained 272.70 Kcal/100 g and 274.26 Kcal/110 g, respectively. It is evident from the results that the addition of chickpea and rice flour contributes to enhance the energy value of ERP bars. Nadeem et al. (2012a); Nadeem et al. (2012b) and Zahra et al. (2014) also reported similar findings as the addition of cereal flour enhances the energy value of nutri‐bars.

### 3.7 SDS‐PAGE of ERP bar's protein

**FIGURE 2:**
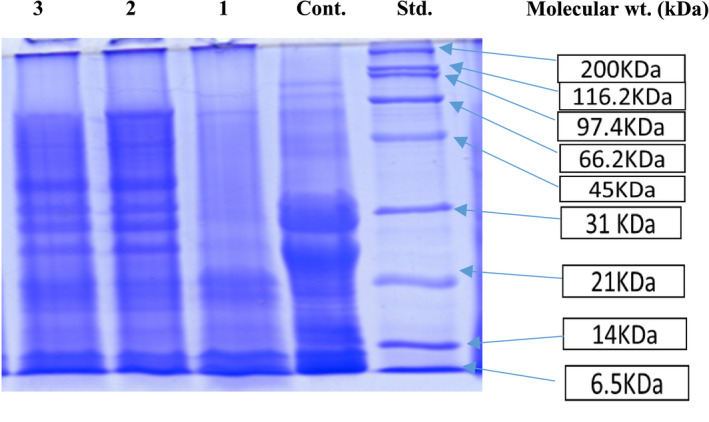
Electrophoretograms of proteins in ERP bars

The electrophoretograms of cheese protein (control), bar #1 (CC and WPI), bar #2 (10% chickpea and roasted gram flour), and bar #3 (5% roasted chickpea and 5% roasted rice flour) are shown in Figure 2 along with the standard marker (200 kDa–6.5 kDa). The control sample showed bands between 6.5 and 31 kDa. The two dense bands at the positions of 31–21 kDa may represent the αs‐CN and β‐CN, respectively. The third very thin band below these two protein bands indicated the κ‐CN **(**Barac et al., 2019 **)**. The band at the position of 31 kDa could be the combination of two proteins (αs1‐CN and αs2‐CN) in the form of αs‐CN as Shori et al. (2020) stated SDS‐PAGE could not separate these two bands because of similar molecular weights. The lower molecular weight protein fraction (below 18 kDa) in cheese could be the product of cheese ripening (Ivens et al., 2017). Panchal et al. (2020) reported that the casein bands fall between 32 and 26 kDa. Similar bands of casein (35 kDa and 25 kDa) are also reported by Afsharnezhad et al. (2019).

The SDS‐PAGE pattern of bar #1 is different from bars #2 & #3. All bars contained 12% WPI and 8% cheese. This portion (8%) of cheese contains around 2% protein, which could be the reason that casein bands are not dominant in all the bar's SDS patterns except very light bands in bar #1. Two bands close to 21 and 14 kDa are also visible in bars # 2 and 3, which could be the protein bands from WPI. The same band was also reported by (Klinchongkon et al. 2019) during their study on WPI. The bands near 21 kDa could be from β‐lactoglobulin (20 kDa) and light chain of immunoglobins (29 kDa), as confirmed by Wang et al. (2020).

The SDS pattern of bars #2 and #3 showed clear four bands above 21 and below 45 kDa of standard. Sofi et al. (2020) reported three bands of 34, 36, and 40 kDa of chickpea proteins in a similar range of present findings. All these protein bands are from chickpeas. No clear visible –80,012 band other than chickpea flour was noticed in bar #3 due to the low rice flour concentration. The concentration of rice in bar #3 was 5% only, which might exhibit 0.3% protein compared to 2% protein in gram flour. In a study, Han et al. (2021) conducted the SDS‐PAGE of chickpea flour and described the six major protein bands with estimated molecular weights of 55, 52.5, 40.2, 38.5, 35.3 23 kDa. Li et al. (2021) reported that the rice protein bands fall between 14 and 66 kDa, which are not visible in the present study due to the low concentration of rice.

### 3.8 ACE‐I inhibitory activity

**FIGURE 3:**
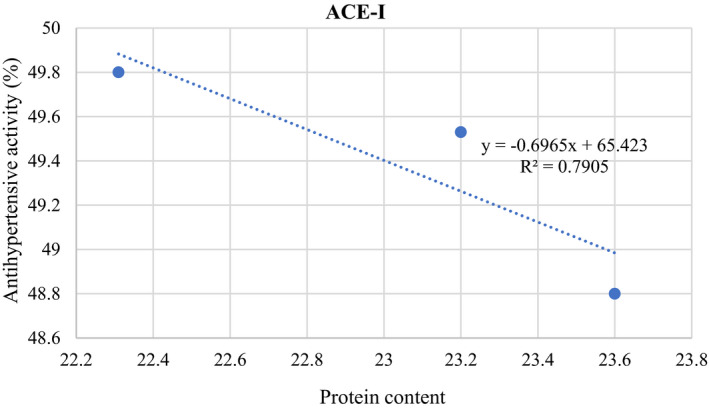
Correlation between protein and ACE‐I antihypertensive activity of ERP bars

The changes in the composition of ERP bars showed a significant impact (*p* < .01) on the ACE‐I. The antihypertensive activity of proteins in cheese (control) and ERP bars #1, #2, and #3 was 31.57 ± 0.5, 49.80 ± 1, 48.80 ± 1, and 49.53% ± 0.8%, respectively. The correlation (Figure 3) between total protein content and ACE‐I activity showed that as the total protein content increased, the ACE‐I activity (*p* > .01) was decreased. The result showed that cheese itself contained less antihypertensive activity as compared to ERP bars. It could be possible that the addition of cheese, WPI, and their mixing with other ingredients of bars increases its antihypertensive activity. Dietary proteins deliver naturally occurring bioactive peptides that are not in an active form in the structure of their native proteins. Different reactions during product formation can affect biologically active peptides and proteins (Tagliazucchi et al., 2020). Cheese and whey‐containing high‐quality proteins and calcium positively impact controlling metabolic disorders and cardiovascular complications. ACE‐I inhibitors reduce the sympathetic outflow by inhibiting the renin–angiotensin system by converting angiotensin I into angiotensin II and preventing the breakdown of bradykinin. At the same time, calcium dilates the large ducts and inhibits the activity of vasoconstrictor hormones such as angiotensin II (Blazic et al., 2017). The ERP bar #2 contained chickpea gram flour along with dates, apricot, cheese, and WPI and showed relatively lower antihypertensive effects compared to bars #1 and #3. This minor difference in bar #2 could be due to the effect of dietary fiber high in chickpea flour and responsible for binding some calcium. It could also increase the total protein content but nonsignificantly reduce the ACE‐I activity (Yegrem, 2021). ACE‐I inhibitory peptides are important in enhancing the vasodilatory peptides, that is, bradykinin, equally decreasing the vasoconstrictor peptides that is, angiotensin II, moderating the BP, and imparting the antihypertensive effect. Smaller peptides/proteins have better antihypertensive activity due to unfolded structure via hydrophobic interaction with active sites (An et al., 2021). Usually, smaller molecular size proteins exhibit more exposure to specific amino acids as compared to their native source that considerably contributes to ACE‐I activity, whereas larger and immense polypeptides may conceal the residues of amino acid that prevents their binding with active sites of ACE‐I protein (Munir et al., 2020).

## 4 CONCLUSIONS

The ERP bars are a healthy snack for athletes in addition to their antihypertensive activities. The consumption of two bars/day containing dates, apricot, cheese, WPI, and chickpea flour provided 40% proteins of their daily recommended dietary allowance. The bars containing only (10% roasted) chickpeas flour were more highly accepted than containing a combination of chickpea and rice flour. Moreover, it also increased the total protein content and diversity of proteins, while antihypertensive was relatively lower than other bars. The protein in ERP bars could be helpful to amplify the stamina building for endurance and resistance training exercise of athletes and preventing them from stress during exercise. These bars' antihypertensive activity can also have a protective effect from cardiovascular diseases.

## Data Availability

The dataset supporting the conclusions of this article is included within the article.
